# Performance of plague rapid diagnostic test compared to bacteriology: a retrospective analysis of the data collected in Madagascar

**DOI:** 10.1186/s12879-020-4812-7

**Published:** 2020-01-30

**Authors:** Minoarisoa Rajerison, Marie Melocco, Voahangy Andrianaivoarimanana, Soloandry Rahajandraibe, Feno Rakotoarimanana, André Spiegel, Maherisoa Ratsitorahina, Laurence Baril

**Affiliations:** 1 Plague Unit, Institut Pasteur de Madagascar, Antananarivo, 101 Madagascar; 2 Epidemiology and Clinical Research Unit, Institut Pasteur de Madagascar, Antananarivo, 101 Madagascar; 3 Central Laboratory for Plague, Ministry of Public Health, Antananarivo, Madagascar; 4 Direction, Institut Pasteur de Madagascar, Antananarivo, 101 Madagascar

**Keywords:** Plague, *Yersinia pestis*, Bacteriological culture, rapid diagnostic test, test performance

## Abstract

**Background:**

Plague is a highly fatal disease caused by *Yersinia pestis*. Late diagnosis hampers disease outcome and effectiveness of control measures, induces death and disease spread. Advance on its diagnosis was the use of lateral flow rapid diagnostic test (RDT).

**Methods:**

We assessed the performance of the plague RDT based on *Y. pestis* F1 antigen detection more than 15 years after its deployment in Madagascar. We compared the RDT with bacteriological culture results, using data from plague notified cases collected during the periods for which both tests were performed independently and systematically.

**Results:**

Used with bubonic plague (BP) patient samples, RDTs had a sensitivity of 100% (95% CI: 99.7–100%), a specificity of 67% (95% CI: 64–70%) with a good agreement between bacteriology and RDT results (86%; κ = 0.70, 95% CI 0.67–0.73). For pneumonic plague (PP), RDT had a sensitivity of 100% (95% CI: 91–100%) and a specificity of 59% (95% CI: 49–68%) and concordance between the bacteriological and plague RDT results was moderate (70%; κ = 0.43, 95% CI 0.32–0.55). Analysis focusing on the 2017–2018 plague season including the unprecedented epidemic of PP showed that RDT used on BP samples still had a sensitivity of 100% (95% CI: 85–100%) and a specificity of 82% (95% CI: 48–98%) with a very good agreement with bacteriology 94% (κ = 0.86, 95% CI 0.67–1); for PP samples, concordance between the bacteriological and plague RDT results was poor (61%; κ = − 0.03, 95% CI -0.17 – 0.10).

**Conclusions:**

RDT performance appeared to be similar for the diagnosis of BP and PP except during the 2017 PP epidemic where RDT performance was low. This RDT, with its good sensitivity on both plague clinical forms during a normal plague season, remained a potential test for alert. Particularly for BP, it may be of great value in the decision process for the initiation of therapy. However, for PP, RDT may deliver false negative results due to inconsistent sample quality. Plague diagnosis could be improved through the development of next generation of RDTs.

## Background

Plague caused by *Yersinia pestis* is endemic in Madagascar, with a seasonal recrudescence between August and April. Plague is a severe bacterial disease with a potentially fatal outcome in the absence of prompt and appropriate antibiotic treatment. Presumptive antibiotic treatment is started after biological sampling if plague is clinically suspected. In Madagascar, biological confirmation is performed by the Central Laboratory for Plague (CLP), a World Health Organization (WHO) Collaborating Center for Plague, which is also hosted at the Plague Unit of the Institut Pasteur de Madagascar (IPM).

Bacteriological culture is the gold standard for the identification of *Y. pestis*. A lateral flow plague rapid diagnostic test (RDT) based on *Y. pestis* F1 antigen detection has been developed, produced, and successfully evaluated to support healthcare agents [1]. In 2006, the WHO revised recommendations for standard plague case definition based on laboratory results, including those of plague RDTs, and the epidemiological context (Additional file 1: Table S1) [2]. RDTs have since been produced by the CLP and distributed with their sampling kits and secured packaging to health care centers (HCCs) every year.

Since 2002, plague RDTs have been routinely used by primary HCCs and hospitals in plague endemic districts of Madagascar and at the CLP for biological samples arriving with a notification form. From 2008 on, they have been used as a screening test: only samples with a positive RDT result have been tested by bacteriological culture. This protocol has been followed until the early 2017–2018 plague season. Indeed, in 2017, Madagascar faced a pneumonic plague (PP) epidemic in two urban areas. A new molecular biology-based diagnostic test was implemented at the CLP and adapted to the epidemic context of PP [3, 4].

After this recent, mainly pneumonic, plague epidemic and because of the changes in laboratory protocols for plague confirmation, we decided to retrospectively analyze the plague RDT performance of both pneumonic and bubonic plague cases. Bubonic plague (BP) cases accounts for more than 80% of notified cases during endemic periods.

## Methods

For Madagascar, all clinically-suspected plague cases, with compatible clinical presentation (fever, sepsis syndrome, lymphadenopathy, and/or acute pneumonitis) and epidemiological features (such as exposure to infected animals or humans and/or evidence of flea bites and/or residence in or travel to a known endemic focus within the previous 10 days), must be reported to the national surveillance system. Clinical and epidemiological data are collected for each patient on a standardized notification form sent to the CLP, accompanied by biological samples (bubo aspirate, sputum, or postmortem organ puncture, as appropriate) collected on sterile swabs and conveyed in Cary-Blair transport medium for RDT and bacteriological diagnosis [5, 6]. Briefly, the bacteriology consists in *Y. pestis* isolation from biological samples either by direct culture on Yersinia selective agar medium (Oxoid Ltd., United Kingdom) or by mouse inoculation followed by culture of spleen-sample from a dead mouse. Culture incubation was done at 26 °C–28 °C for 48 h or more as *Y. pestis* cultures grow slower than other bacteria. Suspected colony was identified by phage lysis test and biochemical identification on API20^E^ strip (bioMérieux, France).

Information from the notification form and diagnostic test results have been recorded in a Microsoft Access® file and were transferred to REDCap (Version 7.6.5) in 2018. A total of 16,221 cases were recorded (January 1, 1998 to April 3, 2019), 76% of which were of BP, 19% PP, and other cases which were categorized as neither PP nor BP (missing information from the clinical form = NP). Table 1 summarizes the available data by period between 1998 and 2019, according to the diagnostic protocol used by the CLP, with the period 1998–2001 being defined as Period 0, as no RTDs were routinely used.

**Table 1:** Biological data according to periods defined by the diagnostic protocols in place (1998–2019)

	Period 0 1998–2001	Period 1 2002–2007	Period 2 2008–2016	Period 3* 2017–2018	Period 4 2018–2019
Description	Before RDT introduction	Implementation of RDT in healthcare centers (HCCs) and the CLP	RDTs used as screening tests: only cases with positive RDT were tested by bacteriology	2017–2018 plague season (including pulmonary plague epidemic)	2018–2019 plague season (August 2018 – April 2019)
Available tests according to protocol	- Bacteriology - Elisa (serology)	- CLP RDT - HCC RDT - Bacteriology	- CLP RDT - HCC RDT - Bacteriology only when RDT positive (CLP or HCC)	- CLP RDT - HCC RDT - Bacteriology - Molecular biology	- CLP RDT - HCC RDT - Bacteriology - Molecular biology
Number of cases	4955 cases (4689 BP, 136 PP)	4221 cases (3728 BP, 260 PP, 233 NP)	4000 cases (3223 BP, 599 PP, 178 NP)	2786 cases (529 BP, 2044 PP, 1 SP**, 212 NP)	259 cases (208 BP, 46 PP, 5 NP)

*Period 3, 2017–2018, is divided into three sub-periods according to the diagnostic protocols (Protocol 1: in 2017, before Sept 11 and after Oct 3: only cases with a positive RDT result were tested by bacteriological culture; Protocol 2: Sept 11 – Oct 3: all PP cases from non-endemic zones were tested by bacteriological culture (independently of the RDT result) and other cases from endemic districts were tested by bacteriological culture only if the RDT results was positive; Protocol 3: From Jan 2018: all notified cases were tested by bacteriological culture)

We assessed RDT performance over time using the bacteriology as a gold standard and RDTs performed at the CLP to avoid bias due to handling effects in HCCs, despite training. We used plague cases for each identified period during which the two tests were performed independently and systematically, i.e. when RDTs were not used as a screening test to direct bacteriological testing. Indeed, only three time periods were used for our analysis: (1) from 2002 to 2007 for all clinical forms (Period 1), (2) between September 11 and October 3, 2017 for PP notified cases from non-endemic zones (Period 3, Protocol 2) and, from January 2018, all notified cases (Period 3, Protocol 3), and (3) 2018–2019 (until April 3rd) for all notified cases (Period 4).

In addition, we excluded cases for which the time of transport was > 7 days, as the time of transport between HCC where the sample is was collected and the CLP where the diagnostic tests were performed can affect the reliability of the results. We also excluded notified cases who declared to have taken antibiotics to which *Y. pestis* is sensitive (aminoglycosides, such as streptomycin and gentamycin; fluoroquinolones, such as ciprofloxacin or levofloxacin; cotrimoxazole, or doxycycline) prior to sample collection, as the use of these antibiotics can lead to false negative results in culture.

Agreement between the RDT and bacteriological culture was assessed by examining the percentage of concordant results and the Kappa coefficient with 95% confidence intervals (95% CI) [7]. RDT sensitivity and specificity were also estimated such as the positive predictive values (PPV) and the negative predictive values (NPV) [8]. SPSS Statistics (version PASW® Statistics 18) and R (version 3.5.3) were used for statistical analysis.

In accordance with the Plague National Control Program, notification of suspected plague cases by HCC remains mandatory and thus no ethics approval was required for the use of the human data (de-identified) for this study.

## Results

We compared the plague RDT and bacteriological culture results, according to the clinical form of plague, for all notified cases during the three time periods for which inclusion and exclusion criteria were satisfied (Table 2).

**Table 2:** Comparison between plague RDT and bacteriological culture results for clinically-suspected plague cases tested at the CLP during the 2002–2007 period and 2017–2018 and 2018–2019 seasons

		2002–2007				2017–2018				2018–2019			
					Total	Bacteriology			Total				Total
		Pos.	Neg.	Miss.		Pos.	Neg.	Miss.		Pos.	Neg.	Miss.	
Bubonic plague													
RDT	Positive	1328	324	0	1652	22	2	0	24	57	23	3	83
	Negative	0	667	0	667	0	9	2	11	0	71	2	73
	Missing	0	0	280	280					0	0	2	2
Total		1328	991	280	2599	22	11	2	35	57	94	7	158
Pneumonic plague													
RDT	Positive	40	45	0	85	1	23	1	25	8	6	1	15
	Negative	0	64	0	64	3	40	6	49	0	27	0	27
	Missing	0	0	27	27	0	0	1	1	0	0	1	1
Total		40	109	27	176	4	63	8	75	8	33	2	43

### Performance of RDTs on bubonic plague cases

During 2002–2007, following the introduction of RDTs as a plague diagnosis tool, the CLP tested 3448 samples of clinically-suspected BP cases, using both RDT and bacteriological culture (Table 2). After applying the exclusion criteria, 2319 cases could be used for RDT performance analyses. Among them, 1328 (57%) were positive and 991 (43%) negative by bacteriological culture. All bacteriologically-positive cases were also positive by RDT. However, among the bacteriologically-negative cases, 324 (33%) were RDT positive. Overall, the concordance between bacteriological and plague RDT results was 86% (κ = 0.70, 95% CI 0.67–0.73, good agreement). Considering bacteriological culture as the gold standard, the RDTs had a sensitivity of 100% (95% CI: 99.7–100%), a specificity of 67% (95% CI: 64–70%).

For the 2017–2018 plague season, the number of notified suspected BP cases that were tested with bacteriological methods independently of the RDT results was much lower, only 33 cases (after exclusions). Among them, 22 (67%) were positive and 11 (33%) negative by bacteriology. Overall, the concordance between the bacteriological and plague RDT results was 94% (κ = 0.86, 95% CI 0.67–1, very good agreement). The RDT still had a sensitivity of 100% (95% CI: 85–100%) and a specificity of 82% (95% CI: 48–98%).

For the 2018–2019 plague season, the concordance between the bacteriological and plague RDT results, based on 151 cases, was 85% (κ = 0.70, 95% CI 0.59–0.81, good agreement) and the RDT also had a sensitivity of 100% (95% CI: 94–100%) and a specificity of 76% (95% CI: 66–84%).

### Performance of RDTs on pneumonic plague cases

Between 2002 and 2007, the CLP received 260 notifications of clinically-suspected PP cases. After the exclusion criteria were applied (Table 2), 149 cases were included in our analysis. Among them, 40 (27%) were positive and 109 (73%) negative by bacteriology. All bacteriologically positive cases were also positive by RDT, whereas 45 (41%) bacteriologically-negative cases were positive by RDT. Overall, the concordance between the bacteriological and plague RDT results was 70% (κ = 0.43, 95% CI 0.32–0.55, moderate agreement) and the RDT had a sensitivity of 100% (95% CI: 91–100%) and a specificity of 59% (95% CI: 49–68%).

For the 2017–2018 plague season, 67 clinically-suspected PP cases were used for analysis, following application of the exclusion criteria. The concordance between the bacteriological and plague RDT results was only 61% (κ = − 0.03, 95% CI -0.17 – 0.10, poor agreement). Only four cases (6%) were positive by bacteriology and, among them, three were negative by the RDT. Therefore, the sensitivity of the RDT was only 25% (with a large 95% CI: 1 to 81%) and specificity 63% (95% CI: 50–75%).

For the 2018–2019 plague season, the concordance between the bacteriological and plague RDT results, based on 41 cases, was 85% (κ = 0.64, 95% CI 0.39–0.89, good agreement) and the RDT had a sensitivity of 100% (95% CI: 63–100%) and a specificity of 82% (95% CI: 65–93%).

A summary of RDT performances according to time periods, including PPV and NPV, are presented in the Table 3.

**Table 3:** Summary of RDT performances according to periods

	Concordance rate	Kappa Coefficient	Sensitivity	Specificity	PPV	NPV
Bubonic plague						
2002–2007	86%	0.70 [0.67–0.73]	100% [99.7–100%]	67% [64–70%]	80% [78–82%]	100% [99–100%]
2017–2018	94%	0.86 [0.67–1.04]	100% [85–100%]	82% [48–98%]	92% [73–99%]	100% [66–100%]
2018–2019	85%	0.70 [0.59–0.81]	100% [94–100%]	76% [66–84%]	71% [60–81%]	100% [95–100%]
Pneumonic plague						
2002–2007	70%	0.43 [0.32–0.55]	100% [91–100%]	59% [49–68%]	47% [36–58%]	100% [94–100%]
2017–2018	61%	- 0.03 [−0.17–0.10]	25% [1–81%]	63% [50–75%]	4% [0–21%]	93% [81–99%]
2018–2019	85%	0.64 [0.39–0.89]	100% [63–100%]	82% [65–93%]	57% [29–82%]	100% [87–100%]

## Discussion

In the early 2000’s, the use of RDTs for BP greatly contributed to the improvement of case management of patients living in remote areas of Madagascar. After more than 15 years of their use for plague diagnosis, we evaluated their performance using bacteriological culture as a gold standard in addition to our recent work [9]. Although positive results obtained using bacteriological techniques were undeniable, other factors can lead to false-negative results: sample quality (saliva rather than sputum), contaminated field samples, preliminary use of antibiotics (usually not reported on the form), and long transport delays to the CLP. Conversely, F1 antigen detection on RDT remained stable within samples contaminated and/or collected after antibiotic use. Nonetheless, bacteriological culture is still the gold standard for *Y. pestis* detection, according to the 2006 WHO recommendations.

The strength of our analysis, in which we included only cases for which the bacteriological culture was performed independently of RDTs, is also a pitfall. Indeed, we ended up with a small number of cases, leading to estimates with large confidence intervals for the 2017–2018 and 2018–2019 plague seasons and cannot exclude false-negative bacteriology sample due to contamination.

Nevertheless, our results are consistent over study time period, except the period 3, and considering bacteriological culture as the gold standard, the sensitivity of the RDT was 100%, confirming the relevance of its use until the availability of a new point-of-care test for remote areas. However, with a specificity of approximately 70 to 80%, RDT cannot be used as a screening test. The sensitivity and specificity of the RDT appear to be similar for both cases of PP and BP, although the confidence intervals were wider for PP. The 2017–2018 season was an exception, RDT was negative for 75% of cases that were positive by bacteriological culture and only a limited number of cases were available for analysis. Such poor performance of the RDT during the response to the PP outbreak stresses the potential variability of RDT performance under extreme circumstances, when HCCs and laboratories are overloaded and work under pressure. In particular, for PP, good-quality sputum samples are much more difficult to obtain than bubo aspirates, emphasizing the need for sputum collection standard process establishment and regular training of healthcare staff in sample collection and the use of RDTs during the inter-plague season. Indeed, during the 2018–2019 season, reinforcement of training and improvement of laboratory condition have positively impacted the specificity results.

Since the urban PP epidemic, the CLP- Plague Unit has systematically carried out a newly-implemented real time PCR (qPCR), targeting *pla* and *caf1* genes. If results from this test are inconclusive, a conventional PCR targeting *pla*, *caf1*, and *inv* genes is performed. The new decision tree has been reported along with all details concerning findings during the epidemic [3]. Comparisons between RDT and bacteriological culture may be skewed by factors which could affect the isolation of *Y. pestis*. Thus, we are currently using a Bayesian approach on the epidemic data to model the conditional dependence between the multiple tests (RDT, bacteriological culture, and molecular biology testing). Given the recent improvement of molecular biology techniques for *Y. pestis* diagnosis, the WHO recommendations may be updated in the near future. Improving all tools for plague diagnosis, including those suitable for point-of-care screening in remote areas remains a research priority to control human plague [10].

## Conclusions

RDT performance appeared to be similar for the diagnosis of BP and PP except during the 2017 PP epidemic in Madagascar where RDT performance was low for PP. This RDT, with its good sensitivity on both plague clinical form during a normal plague season, remained a potential test for alert in plague endemic areas. Particularly for BP, it may be of great value in the decision process for the initiation of therapy. However, for PP, RDT may deliver false negative results due to inconsistent sample quality (prone to errors). A combination with other rapid tests, microscopy or a new generation of RDT, could address the issue of its moderate specificity. Plague diagnosis could be improved through the development and implementation of next generation of RDTs.

## Supplementary information

**Additional file 1:** **Table S1.** Case definition, based on the 2006 WHO recommendations, using three diagnostic tests performed at the IPM: rapid diagnostic tests (RDT), molecular biology testing, and bacteriological culture

## Data Availability

All data generated or analyzed during this study are included in this published article.

## Abbreviations

BP: Bubonic plague
CI: Confidence Interval
CLP: Central Laboratory for Plague
HCCs: Health Care Centers
NPV: Negative Predictive Values
PP: Pneumonic plague
PPV: Positive Predictive Values
RDT: Rapid Diagnostic Test
REDCap: Research Electronic Data Capture
SP: Septicemic Plague
