# Supplementary material for: Performance of plague rapid diagnostic test compared to bacteriology: a retrospective analysis of the data collected in Madagascar
Source: BMC Infect Dis. 2020 Jan 30;20:90. doi: 10.1186/s12879-020-4812-7 (PMC6993518; doi:10.1186/s12879-020-4812-7)
Supplement: Supplementary file 1 — Additional file 1: Table S1. Case definition, based on the 2006 WHO recommendations, using three diagnostic tests performed at the IPM: rapid diagnostic tests (RDT), molecular biology testing, and bacteriological culture [file 12879_2020_4812_MOESM1_ESM.docx]

**Additional file 1**

Table S1. Case definition, based on the 2006 WHO recommendations, using three diagnostic tests performed at the IPM: rapid diagnostic tests (RDT), molecular biology testing, and bacteriological culture.

| **Class** | **Definition** |
| --- | --- |
| Suspected cases | All clinically-suspected plague cases that meet the clinical and epidemiological criteria according to WHO recommendations* |
| Probable cases | Clinically-suspected cases with |
|  | positive RDT or positive molecular biology |
|  | and |
|  | negative culture or not performed |
| Confirmed cases | Clinically-suspected cases with |
|  | positive RDT and positive molecular biology |
|  | or |
|  | positive culture |

* Compatible clinical presentation (fever, sepsis syndrome, lymphadenopathy, and/or acute pneumonitis) and epidemiological features (such as exposure to infected animals or humans and/or evidence of flea bites and/or residence in or travel to a known endemic focus within the previous 10 days).
